# Next-generation CAR-T cell therapy against cancer: precision engineering, programmable immunity, and emerging clinical frontiers

**DOI:** 10.1186/s43046-026-00415-1

**Published:** 2026-09-29

**Authors:** Faizan-e- Mustaffa, Muhammad Shahbaz Aslam, Muhammad Junaid

**Affiliations:** 1 University of the Punjab, Lahore, Pakistan; 2 Khwaja Fareed University of Engineering and Information Technology, Rahim Yar Khan, Pakistan

**Keywords:** Chimeric antigen receptor T cells (CAR-T), Cancer immunotherapy, Genome editing, Synthetic biology, Precision immunotherapy

## Abstract

While chimeric antigen receptor T-cell (CAR-T) therapy has revolutionized the treatment of hematological malignancies, there are significant hurdles to overcome, such as antigen escape, T-cell exhaustion, limited persistence in the body, severe toxicities, manufacturing complexity, high costs, and low effectiveness in the treatment of solid tumors. There are many reviews describing novel CAR-T technologies, the evidence strength, clinical maturity and unmet translational risks of each are largely included in those reviews. This review highlights the ways in which next-generation engineering increases the safety, efficacy and programmability of CAR-T. The primary and authoritative clinical, regulatory and preclinical evidence on recent advances from 2022 to 2026 was carefully evaluated, including the quality and quality control of the evidence for each strategy. New innovations are Armored CARs, dual-target and logic-gated/synNotch systems, universal and switchable platforms, CRISPR/Cas9 and base/prime editing, allogeneic CAR-Ts, non-viral manufacturing, and lipid-nanoparticles in vivo CAR-T generation. Additionally, the engineering and combination approaches for the treatment of solid tumors with checkpoint inhibitors, radiotherapy, oncolytic viruses, nanotechnology, and artificial intelligence are discussed. An evidence-to-maturity framework separates the clinically proven methods from the emerging technologies and highlights some of the critical translation hurdles related to safety, immunogenicity, durability, manufacturing and scale. Precision Immunotherapy is becoming more and more possible with the next generation CAR-T engineering, however the validation and translational optimization of these cells is dominant to their eventual clinical use.

**Graphical abstract:**

**Figure:**
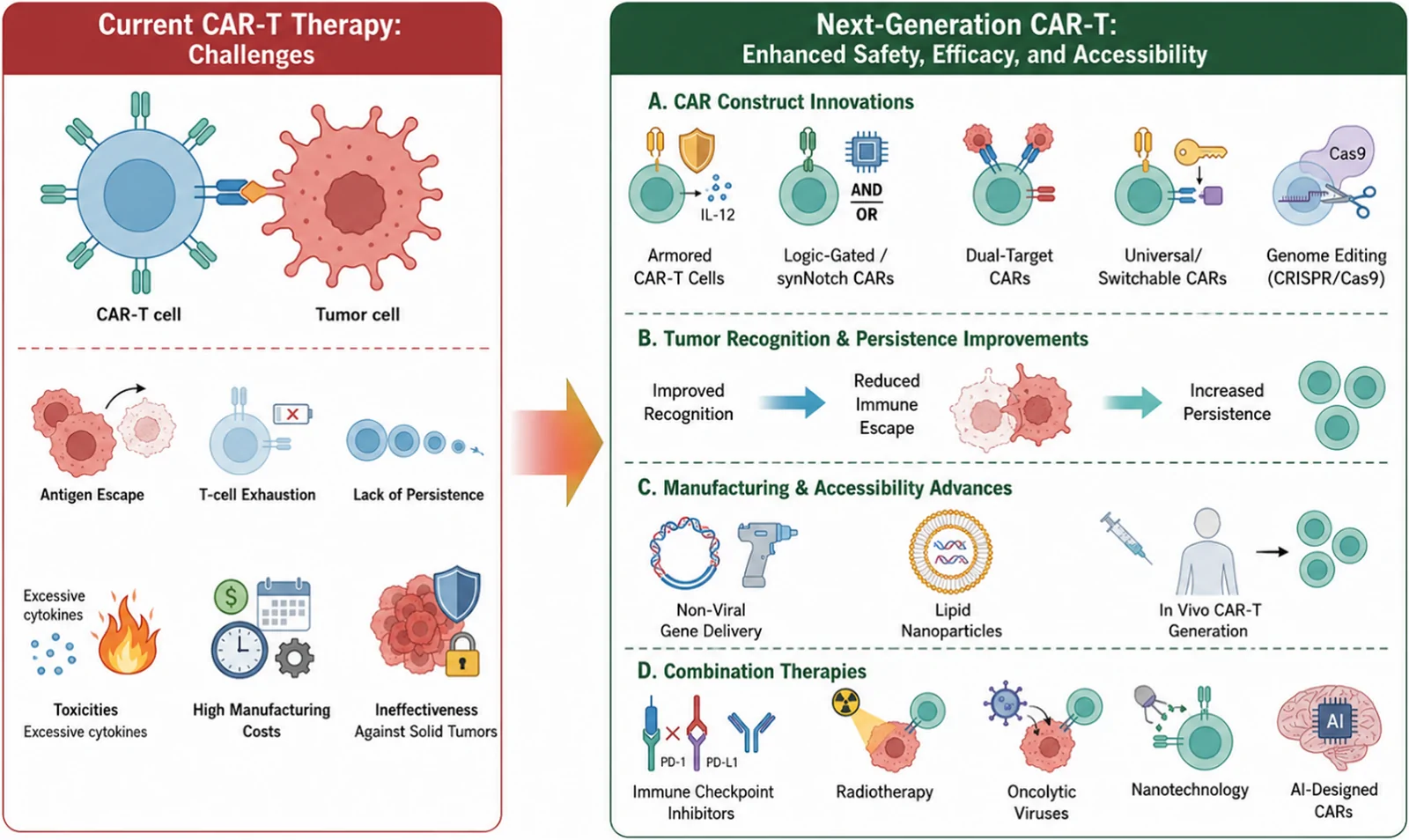

## Introduction

Despite major advances in surgery, radiotherapy, chemotherapy, targeted therapies and immune checkpoint inhibitors, one of the leading causes of death in the world is still cancer [1]. Although these traditional therapeutic approaches have contributed to major advances in survival for patients with many malignancies, there remain many advanced and relapsed malignancies that are incurable. This occurs due to the different subtypes of the tumor, acquired drug resistance, immune evasion and metastatic progression [2]. In the last decade, the therapeutic efficacy of adoptive cell transfer has transformed by employing the patient’s immune system to eliminate tumor cells [3]. These strategies have made chimeric antigen receptor (CAR)-T cell therapy one of the most successful precision cancer immunotherapies to revolutionize the treatment of all hematological malignancies especially those that are unmanageable or relapsed [4].

CAR-T cells are genetically modified lymphocytes that express artificial receptors. These receptors are able to recognize surface antigens associated with tumors in a major histocompatibility complex (MHC)-independent way. Unlike typical T-cell receptors, CARs structure consists of the antigen specificity of a mAb and the T-cell activation mechanism that allows them to directly kill malignant cells. This type of immunity is independent of MHC, and circumvents multiple immune-evasion strategies exploited by tumors, and has strong cytotoxic activity even in antigen presentation deficient patients [5]. Because of the clinical translation of the technology, a number of CAR-T products targeting CD19 and B-cell maturation antigen (BCMA) has been approved by regulators. This has resulted in unprecedented success in terms of response rates and long-term remissions in B-cell acute lymphoblastic leukaemia (ALL), as well as a variety of B-cell malignancies such as diffuse large B-cell lymphoma, mantle cell lymphoma, follicular lymphoma and multiple myeloma [6].

Conventional CAR-T immunotherapies have achieved good clinical outcomes, but they have yet to fully realize their therapeutic potential. Antigen loss, antigen heterogeneity, lineage switching and T-cell exhaustion are potential mechanisms for relapse after first-line remission [7, 8]. Moreover, the solid malignancy tumor microenvironment (TME) plays a role in the immunosuppressive environment. Physical stromal barriers, hypoxia, metabolic competition, immunosuppressive cytokines, regulatory immune cells and inhibitory checkpoint signaling are just a few examples [9]. All these factors severely limit the infiltration of CAR-T. Moreover, CAR-T’s persistence and cytotoxicity are limited and access to treatment is restricted due to cytokine release (CRS), immune effector cell-associated neurotoxicity syndrome (ICANS), slow manufacturing process, personalized manufacturing, and high cost of treatment [10, 11]. Improved efficacy with acceptable safety is a critical need for engineering solutions.

As a result, the CAR-T therapy has passed on to a phase of engineering accuracy instead of modifying incremental receptors [12]. Recent advances in synthetic biology, systems immunology, and genome engineering have made the development of programmable immune cells possible, able to sense multiple signals associated with tumor cells, include Boolean logic circuits, respond selectively to specific signals in the microenvironment, and modulate their functional state [13]. The newly developed CAR platforms designed by scientists including logic gated receptors, synNotch, cytokine armored constructs, cytokine-activating systems, engineering chemokine receptors, metabolic rewiring and synthetic gene circuits have the highest tumor specificity, persistence and do not exhaust [14]. Meanwhile, the precision genome editing techniques like CRISPR-Cas9 and base and prime editing have enabled the development of improved and more immunologically non-threatening universal allogeneic CAR T cells [15].

A second key trend is the transition from a single-use autologous therapy to a mass market cellular medicine. Universal, healthy donor derived generated products; manufacturing using induced pluripotent stem cells (iPSCs); generating from natural killer (NK) cells; generating from macrophages; other immune cell populations that do not lead to manufacturing delays; manufacturing that is less expensive [16, 17]. In vivo CAR-T generation is now possible by means of targeted lipid nanoparticles and other non-viral delivery technologies [18]. Genetically reprogrammed endogenous T cells in patients may even avoid the need for cell manufacture outside the body [18]. These advances are expanding the potential uses of CAR therapies beyond hematological malignancies to solid tumors, auto-immune disorders, fibrosis, infectious diseases and other immune-mediated diseases, offering sign into the development of CAR technology as a powerful platform for programmable precision medicine, not just as an anticancer therapy [19].

This review focuses on the innovations in the next-generation CAR-T cell therapy and published literature from the year 2022–2026 and the recent advancements reported in the year 2025–2026. We review the evolution of CAR architecture/engineering, in vivo CAR-T manufacturing technologies, universal and in vivo platforms to overcome the tumor microenvironment, combination therapy, CAR-T design approaches using artificial intelligence, and recent clinical developments. In this review, we summarize the various incremental improvements that have been achieved by these new generation CAR-T technologies, which will drive the development of safer, more effective, scalable and programmable cellular therapies for cancer immunotherapy.

### The evolution of CAR-T cell therapy: from conventional receptors to programmable cellular therapeutics

CAR-T cell therapy is an amazing success story that is being recognized worldwide. It has been more than 30 years since the very first CAR construct was proposed. The CAR-T cells have become progressively more complex over the years with improvements in receptor architecture, intracellular signaling, and cellular engineering [20]. The first generations were mainly related to T-cell activation and persistence. More synthetic biology, Boolean logic circuitry, genome editing, cytokine engineering, and universal targeting, for more dynamic responses to complex tumor environments, are integrated into the newer CAR platforms [13]. Therefore, evolution of CARs should not only be considered as the development of new and improved CARs but also the evolution of tailored cells of the immune system that can independently detect, decide and destroy the tumor [12].

CARs recognize cell surface antigen without its presentation by the major histocompatibility complex (MHC) in contrast to the conventional T cell receptor (TCR). This property makes it possible to avoid the downregulation of MHC molecules on the surface of tumor cells, enabling direct recognition of tumor-associated antigen [20]. A CAR is made of a single-chain variable fragment (scFv), a hinge region, a transmembrane domain and one or more intracellular signaling domains [21]. Each CAR (scFv) has an extracellular antigen-binding domain, a hinge region that affects receptor flexibility and antigen binding and a transmembrane region that imparts stability to the receptor [12]. A CAR’s efficacy is dictated by one or more intracellular signaling domains needed to activate or mediate the CAR’s effector function. Optimizing each component of a CAR, such as optimizing the affinity of the scFv, hinge length, transmembrane structure, and the signaling motifs, can all impact receptor sensitivity, persistence, cytokine production and toxicity; therefore, structural engineering is a key focus for next generation CAR design [22]. A summary of different generations of CAR-T cells is given in Table 1.

**Table 1:** Details and status of different CAR-T generation

Generation	Defining Feature	Principal Clinical/Biological Consequence	Status As of 2026	References
First generation	CD3ζ signaling domain only, no co-stimulation	Antigen-directed killing but poor persistence; T cells became anergic/exhausted without co-stimulation; disappointing early trials	Historical; superseded	[23–25]
Second generation	Single co-stimulatory domain (CD28 or 4-1BB) added to CD3ζ	Markedly improved proliferation, cytokine production, metabolic fitness, and persistence; CD28 favors rapid strong effector responses with shorter persistence, 4-1BB favors mitochondrial fitness and memory formation	Current clinical standard; basis of all approved CD19/BCMA products	[26–28]
Third generation	Two co-stimulatory domains (e.g., CD28 + 4-1BB or OX40)	Increased cytokine production in preclinical models but no consistent clinical efficacy benefit over second generation, with greater toxicity risk	Largely not adopted clinically; second generation remains the benchmark	[23, 29, 30]
Fourth generation (TRUCKs/armored CARs)	Activation-dependent secretion of immunostimulatory cytokines (IL-12, IL-15, IL-18, IL-21, GM-CSF)	Local immune activation of the tumor microenvironment without systemic cytokine toxicity; a leading strategy for solid-tumor resistance	Multiple candidates in early-to-mid clinical development	[31–33]
Fifth generation	Truncated cytokine-receptor motifs (e.g., IL-2Rβ with STAT3/STAT5 binding sites) fused with CD3ζ and a co-stimulatory domain	More physiological, cytokine-independent activation; improved persistence and memory differentiation in preclinical models	Early clinical development	[34, 35]

Recent studies shows that the generational classification of CAR-T cells is no longer suitable to describe the complexity of the current changes in their receptors. The present innovation relates to the upcoming generation of receptors rather than to the precision modification, which is the integration of modifications that enable immune cells to sense more than one environmental stimulus, to process biological information and to perform context-specific therapeutic responses [14, 36]. CARs in modern CAR platforms are equipped with logic gated receptors, synNotch circuits, inhibitory CARs (iCARs), adaptor-mediated universal CARs, drug-inducible ON-switch receptors, nanobody and DARPin based recognition domains, genome-edited allogeneic products, metabolic rewiring and programmable gene circuits. These technologies have enabled selective activation within the tumor, reduced off-target toxicity, the ability to overcome antigen heterogeneity, and increase the persistence in immunosuppressive environments. Overall, these innovations are transforming CAR-T therapy from receptor-based to cellular-based, setting the stage for the next generation in precision [20, 37].

Given that head-to-head trials are not routinely conducted between different generations of CARs, it is important to understand that newer designs have demonstrated superior clinical results. In one “within-patient” trial, patients were given a second-generation and a third-generation CD19 CAR-T side-by-side. The third-generation cells grew more and lived for longer in the bloodstream. This more robust expansion did not necessarily mean a better remission rate however when compared to second generation products that are already being used in the clinic [12, 38]. Other 3rd generation constructs with additional signaling or regulatory domains have shown comparable levels of response to second generation constructs, and once more there is no convincing evidence of better efficacy [12, 38]. The evidence for 4th generation (armored) and 5th generation (cytokine-receptor) CARs is limited, and most of the evidence for the newer designs comes from small, single arm early-phase trials which cannot prove that a new-generation CAR is superior to the standard of care [38]. Hence, single-arm information on next-generation CAR should be approached carefully, and not as evidence of superiority, until randomized or matched comparative studies are available [12, 38].

### Biological and clinical limitations of conventional CAR-T therapy

By addressing the biological, clinical, and manufacturing hurdles associated with conventional CAR-T therapy, next-generation approaches have the ability to expand the clinical success of CAR-T cells beyond hematological malignancies to a broader range of solid tumors[39]. The effectiveness of CAR-T cells as living therapies does not merely depend upon antigen recognition, but rather on sustained proliferation, persistence, trafficking, and adaptation [40]. Thus, if a CAR-T therapy doesn’t work, it is not due to a single issue but often due to combination of various issues. Next-generation engineering strategies are designed to tackle these barriers simultaneously, instead of just using stronger activation signals. Relapse is mainly due to antigen loss and tumor heterogeneity. Immunological pressure can cause down-regulation of the antigen, acquisition of escape mutations, lineage switching, or the loss of the antigen. This is especially difficult in solid tumors where the antigens of each region of the tumor are different [41]. Based on these observations, CAR systems have been developed that target more than one antigen (dual-target, tandem and multispecific CAR systems), or that target only antigens that are present in the cell (logic-gated CAR) [42].

Progressive T-cell exhaustion is another important issue. Exposure to antigen and a continuous inflammatory signal will result in a gradual decrease in CAR-T proliferation, cytokine production, and killing capacity. Furthermore, there will be a progressive increase in inhibitory receptor expression (PD-1, LAG-3, TIM-3 and TIGIT). The continuous stimulation also leads to an increase of terminal differentiation and cellular senescence, which in turn impairs the production of long-lived memory T cells for a stable immune surveillance. The tiredness is not just an issue of antigen exposure; it also involves transcriptional/epigenetic and metabolic reprogramming, and thus new platforms are increasingly geared towards re-wiring checkpoints, armoring cytokines and metabolic engineering to maintain stem-like memory phenotypes and extend anti-tumor activity [43]. Another obstacle is the immunosuppressive tumor microenvironment (TME) of solid tumors [44]. The dense environment caused by the extracellular matrix, abnormal blood vessels, increased interstitial pressure, and presence of cancer-associated fibroblasts leads to restricted infiltration of CAR-T cells. Once they arrive in the T cells, other barriers reduce their effect. Examples include regulatory T cells, myeloid-derived suppressor cells, inhibitory cytokines (e.g., TGF-β and IL-10), and checkpoint ligands (e.g., PD-L1) [45]. Limitation in tumor homing has led to the engineering of new chemokine receptors, such as CCR2, CXCR1, CXCR2, and CXCR6, to enhance tumor trafficking and infiltration, which has been promoted by poorly matched chemokine signaling [46, 47].

**Fig. 1:**
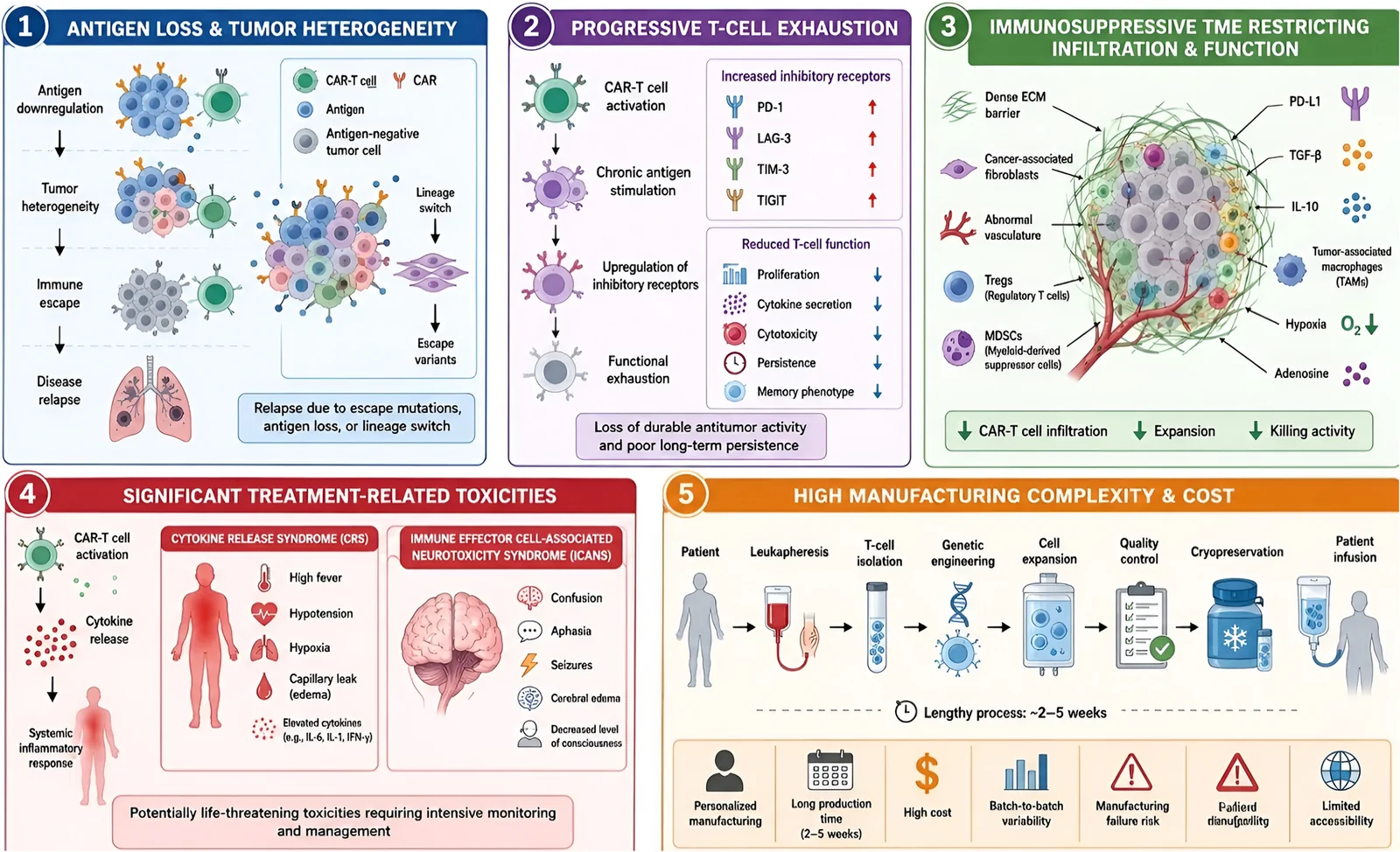
Biological and clinical limitations of conventional CAR-T therapy

In general, there are still significant clinical barriers, such as toxicities associated with treatment and manufacturing challenges. The severity and life-threatening potential of cytokine release syndrome (CRS) and immune effector cell-associated neurotoxicity syndrome (ICANS) [48]. Individualized autologous manufacturing leukapheresis, viral transduction, ex vivo expansion and quality-release testing takes weeks to perform. Because of their extremely high cost, availability is limited to specialized centers, creating inequalities in access [49]. Several factors have a single impact on the success of the manufacturing processes, including previous chemotherapy treatment, the quality of lymphocytes and the disease burden [50, 51]. In heavily pretreated patients, however, product variability, as well as total manufacturing failure, still exist. All these constraints suggest that the currently marketed second-generation CAR-Ts are not the final answer, but rather a stepping stone to the programmable, genome edited and universal platforms [52]. Figure 1 provides summary of limitations of conventional CAR-T cell therapy.

### Precision engineering and programmable control of next-generation CAR-T cells

Today’s CAR-T engineering has gone beyond co-stimulatory domains addition. Contemporary CAR-T engineering professionals always put an extra effort into titrating every aspect of receptor and cellular function. Antigen recognition, the strength of signal, persistence, trafficking and safety have all been discussed. So, with that, CAR-Ts can be turned on or off when needed as context-dependent therapeutics, not indiscriminate killers.

The specific and sensitive interactions with the antigen are largely determined by the antigen-binding domain in the receptor, which usually comprises a single-chain variable fragment (scFv) in the extracellular part of the receptor. Too high affinity will unexpectedly result in tonic signaling and early exhaustion, making it preferable to have the so-called intermediate-affinity binders to achieve serial killing of tumor cells and thus maintain persistence, which is the design of modern ones [53]. Alternative scaffolds are nanobodies, which are designed ankyrin repeat proteins (DARPins), and monobodies. These proteins are smaller, less immunogenic, more thermally stable and more easily assembled into multispecific or modular receptors than traditional scaffold protein options [54]. These benefits render them appealing for the treatment of heterogeneous solid tumors. The passive domains like hinge and transmembrane regions are now known to influence immune synapse formation and activation threshold. The control center of the receptor is its intracellular signaling domain. CD28 promotes rapid, strong effector response, at the expense of the rapid terminal differentiation, while 4-1BB promotes mitochondrial fitness, memory formation, and long-term persistence [29, 55]. In an alternative approach to maximizing signal strength, engineers are working on tuning immunoreceptor tyrosine-based activation motifs (ITAMs), phosphorylation sites, and introducing other co-stimulatory molecules like ICOS, OX40, and CD27 to control potency versus durability. Genome editing can be used to eliminate genes that inhibit the tumor response (such as PD-1 or endogenous TCR), reprogram metabolic pathways to the hypoxic tumor environment, alter and modify chemokine receptors to optimize tumor homing, or insert synthetic transcription circuits that condition the expression of genes to disease-specific signals [56, 57]. All these improvements combine to make CAR-T cells not just cytotoxic but intelligent therapeutic agents that respond to their environment and act in a controlled way [58].

As the antigen heterogenity or off-tumor toxicity is not resolved by receptor optimization alone, the field has adopted the synthetic biology “logic gates” which require CAR-T cells to integrate more than one signal for killing [42, 59]. Discrimination against normal tissue, which rarely co-expresses two antigens, can be achieved by a CAR that signals on simultaneous recognition of two antigens (an AND-gate CAR) which can be obtained by using a split-signaling receptor or a sequential activation circuit [60]. This approach is already moving to early trials in glioblastoma, pancreatic cancer and mesothelioma. To prevent the development of antigen-negative relapses, CARs that will function as OR-gates are built as tandem constructs or bicistronic or dual-receptor constructs that are triggered on recognition of either of the two antigens. OR-gate CARs can be applied in the case of heterogeneous malignancies or hematologic malignancies [61, 62]. The NOT-gate or inhibitory CARs (iCARs) are the exact opposite. They include both a recognition domain for healthy-tissue antigen and inhibitory elements from PD-1 or CTLA-4 to actively inhibit activation when the healthy tissue is encountered in conjunction with the tumor antigen [63].

One of the most versatile programmable platforms that have emerged to date is the synthetic Notch (synNotch) receptor. The synNotch receptor has a different functionality compared to other CARs as it is not directly stimulating the activities of T-cells. Rather, after the recognition of the antigen, the first molecule to get cut off is a transcription factor that will turn on the expression of the second CAR, a cytokine or a checkpoint inhibitor in the proper tumor setting [12, 64]. This is done and thereby provides a very specific AND-gate, which localizes immunomodulatory molecules to the tumor site only [65]. Besides these genetically defined circuits, drug-inducible “ON-switch” CARs only assemble into a functional receptor upon the presence of a small molecule, providing temporal, pharmacologic control over CAR-T, including the ability to pause treatment when toxicity occurs [66, 67]. These systems are a step toward the “smart” immune cells that can be programmed for a context-dependent multi-signal decision-making process rather than a state of constitutive cytotoxic activity.

### Universal and off-the-shelf CAR-T cell therapies

Production of CAR-T agents is personalized and is thus one of the key challenges to making CAR-T more widely available. The leukapheresis, ex vivo modification, expansion and release testing procedures require weeks and patients can progress or become ineligible during this period, and the quality of the product is highly dependent on the ability of the patient own, often highly pretreated lymphocytes [68]. CAR-T platforms that can be said to be “universal” or “off-the-shelf” are produced in advance and kept in stock from healthy donors [52]. These CAR-T platforms feature the ability to overcome these limitations and provide consistency, scalability, and cost improvements. In general, healthy donor products tend to have a greater proliferative capacity and antitumor activity than heavily pretreated patient products because they contain more functional naive and memory T cells [69, 70]. Standardized biologic medicines also enable the more stringent quality control which is available in centralized manufacturing [71].

In general, allogeneic CAR-T cells from healthy donors exhibit superior proliferation and cytotoxicity properties compared to autologous CAR-T cells [72]. But the chances are that there is a risk of graft-versus-host disease (GVHD) due to the presence of native T-cell receptors (TCRs). There can also be recognition and rejection by the host immune of donor HLA molecules. Again, the problems are solved using multiplex genome editing. The goal of the TRAC locus deletion is to eliminate the endogenous TCR to avoid GVHD recognition [56]. Both the B2M and HLA class I knockout decreases recognition by the host cytotoxic T cells [73]. Another protection strategy is an increase in expression of non-classical molecules (e.g. HLA-E, HLA-G) and anti-phagocytic molecules (e.g. CD47) for protection against elimination by NK cells [74, 75]. Thus, recent clinical and translational studies highlight the need to not only produce an allogeneic CAR-T cell, but to ensure a balance between prevention of GVHD, evasion of the host immune system, resistance to NK cells, effective anti-tumor activity, and long-term persistence. For instance, multiplex disruption of TRAC in combination with HLA class I-related pathways has shown that targeting tumor cells while simultaneously preventing GVHD does not result in complete loss of the CAR-T antitumor effects; however, HLA disruption also increases the recognition of NK cells, highlighting the potential pitfalls of multiplexing universal-cell targeting [76, 77]. These results have motivated the creation of increasingly complex “stealth” CAR-T designs incorporating combinations of TCR deletion, HLA engineering, NK-cell inhibitory signals, and other immune-modulatory modifications [76, 78, 79].

The alternative path to universality is without permanent redesign of receptors. T cells can be modified to express a fixed universal receptor that can recognize an adaptor molecule. This molecule then attaches to the tumor antigen. Exchange of adaptor changes specificity without the need to re-engineer the cell. Furthermore, it allows for multi-antigen targeting either sequentially or simultaneously [80]. Last, it allows the clinician to easily remove the adaptor and stop CAR-T activity, offering a safety feature [81]. Another pathway to increased standardization and manufacturing scale is via induced pluripotent stem cells (iPSCs), where the endogenous TCRs, HLA molecules and checkpoint receptors can be deleted in a complex multiplex edit at the pluripotent level and subsequently differentiated into genetically homogenous, precision engineered immune-cell populations [82, 83].

Furthermore, the concept of universality is becoming more widespread than T cells. CAR-NK cells have a low risk of inducing GVHD [84]. They secrete decreased inflammatory cytokines that are linked to ICANS and CRS. They also have intrinsic mechanisms for killing antigen-lacking tumor cells that are partially off-targeted by CARs [85]. Furthermore, CAR-macrophages and other engineered myeloid cells are being investigated for their ability to infiltrate dense stromal tissue and remodel the tumor microenvironment [86]. Universal products are already under trials, and initial results indicate they are safe and have anti-tumor activity, but it will be determined if they will remain long-term. These platforms are a change from personalized manufacturing to off-the-shelf cellular medicines to use in the clinic [87, 88].

#### Next-generation CAR immune-cell platforms beyond CAR-T: CAR-NK, CAR-NKT, CAR-γδ T, and CAR-macrophages

From the review, it appears that so far, the use of CAR-T cells has proven to be successful on target cancer cells. There are other engineered cells with CAR that are currently being developed and small scale cultured, in addition to CAR-T. CAR-NK cells, discussed above, kill tumor cells in CAR-dependent and natural, antibody-independent fashion, possess a low risk for GVHD (graft-vs-host-disease), since they are not dependent on a rearranged T-cell receptor [89, 90]. CAR-NK cells can be produced from cord blood, peripheral blood or iPSCs and may be available as a ready-to-use product [90, 91]. CAR-NKT cells are T cells that are engineered to overcome two limitations of conventional CAR-T cells. First, they express an HLA-unbinding invariant T cell receptor (iTCR) and are therefore not able to cause GVHD even in the absence of the iTCR knockout. Second, due to their innate nature, they keep high-affinity innate tumor trafficking and cytotoxic activity, through their CAR and endogenous receptors. CAR-NKT cells can therefore exploit multiple mechanisms of tumor recognition, including CAR-mediated recognition together with endogenous TCR- and NK-receptor-associated functions. With the clinical relevance of low frequency of NKT cells in circulating lymphocytes, allogenic CAR-NKT cells can now be differentiated by clinically relevant methods from hematopoietic stem and progenitor cells, leading to the production of banked products enhanced with IL-15 and manufactured at scale [92].

Preclinical activity of these allogeneic CAR-NKT platforms has been promising in many challenging cancers. Hematopoietic stem and progenitor cell-derived CD33-directed CAR-NKT cells have been shown to be able to infiltrate and kill malignant cells within the bone marrow, an area that is difficult to access by CAR-T cells for models of myeloid malignancy [92]. Allogeneic CAR-NKT cells can simultaneously attack tumor cells and the immunosuppressive tumor microenvironment, as two therapeutic barriers specific to solid tumors have been identified to hamper CAR-T efficacy in the context of ovarian cancer [93, 94]. Triple-negative breast cancer (TNBC) is a therapy-resistant form of the disease and mesothelin-targeted allogeneic CAR-NKT cells caused a robust anti-tumor response in this model of TNBC. It was effective in primary tumor and metastatic models, without graft-versus-host activity. Within this study, it showed a dual effect of CAR-mediated killing, natural NK-like cytotoxicity and direct targeting of CD1d expressing immunosuppressive cells in the tumor environment [95]. When combined, these research findings support CAR-NKT cells as one of the more hospital-ready “beyond CAR-T” tools for curing solid cancers. Like CAR-NK cells, most evidence in support of these therapies to date remains preclinical or early-phase, however, with bigger control trials still needed to verify durable clinical benefit.

Two more platforms expand CARs to other innate and myeloid lineages. Both the CAR-directed mechanism of recognition, as well as the innate, MHC-independent recognition ability of γδ T cells to stress-induced ligands on tumor targets, are two pathways by which CAR-γδ T cells can recognize their target. This means that CAR-γδ T cells have two mechanisms to target tumor cells and they also have natural allogeneic compatibility that αβ CAR-T cells lack. CAR-macrophages (CAR-M) take advantage of the natural property of macrophages to penetrate into hypoxic dense tumor tissue and remodel the stroma. In addition to their phagocytic killing ability, CAR-Ms can eliminate immunosuppressive tumor-associated macrophages and cross-prime adaptive T-cell responses, these two are both potentially useful to transform immunologically “cold” solid tumors to “hot” ones. Both platforms have shown synergic antitumor activity which is generally associated with reduced toxicity in preclinical models, with conventional CAR-T cells. However, neither platform has been further developed in the clinic than the CAR-NK or CAR-NKT platforms. However, a challenge for clinical manufacture for CAR-M in particular is that primary macrophages are technically difficult to transduce and expand, which is a big hurdle to translation [96].

In other words, the advantages of these different CAR-immune-cell platforms are that they generally sacrifice some of the capacity for expansion and long-term persistence of the T cell, but introduce less risk of GVHD and cytokine driven toxicity, a capacity for killing antigen negative tumor cells through non-CAR mechanisms, and in most cases, a simpler allogeneic, off-the-shelf manufacturing process [90, 97]. Among the CAR platforms, CAR-NK and CAR-NKT are the most advanced platforms available clinically. For this reason, they have several trials in the early phase that have been completed or are still in progress [90, 98]. Meanwhile, the CAR-γδ T and CAR-M platforms remain mostly within the pre-clinical stage of development [99]. No one of these platforms has been directly compared to CAR-T cells in a randomized trial, and hence, their clinical utility over manufacturing and safety benefit remains unclear [91, 98].

### Genome editing technologies for next-generation car-t cells

**Fig. 2:**
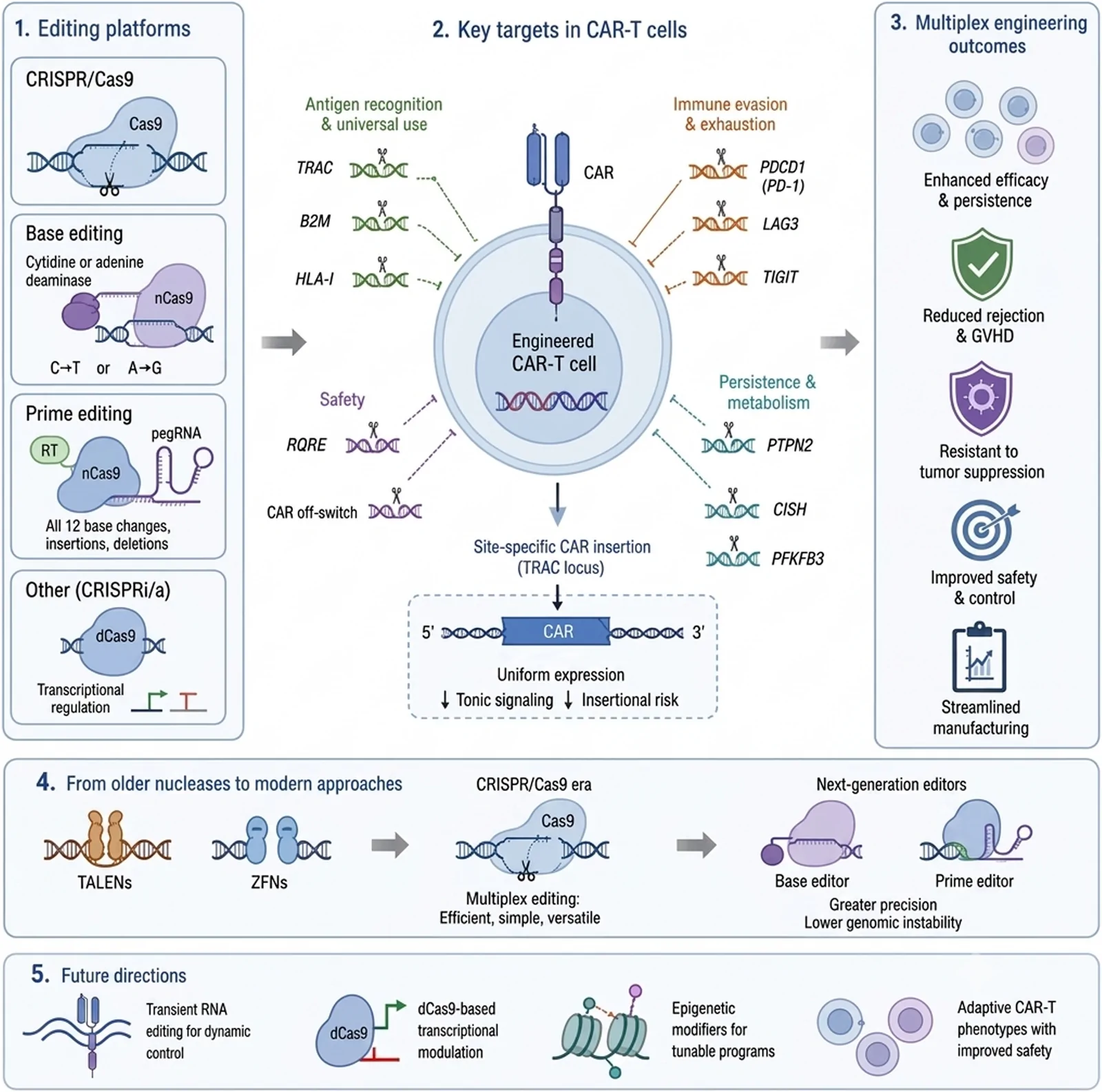
Overview of genome editing technologies and genetic engineering strategies for next-generation CAR-T cells, highlighting editing platforms, key gene targets, therapeutic outcomes, and future directions

The ability to make specific genetic modifications to the genes controlling antigen recognition, immune evasion, persistence, metabolism, and safety in the next-generation CAR-T has come into the forefront of CAR-T design. With targeted genome editing, i.e. making precise edits to endogenous genes while sparing most of the genome, engineers can move from single-gene addition to broad redesign of T-cell biology [56]. Figure 2 summarizes the major genome editing technologies, key genetic targets, therapeutic outcomes, and emerging engineering strategies that are driving the development of next-generation CAR-T cells.

Although non-homologous end joining or homology-directed repair could occur to fix the DSB, CRISPR/Cas9 is the preferred system because of its simplicity and multiplex ability [15]. TRAC deletion to prevent GVHD in universal products, B2M or HLA disruption to reduce host rejection, and PDCD1 (PD-1) knockout to blunt tumor-mediated suppression in CAR-T engineering have been made possible by CRISPR/Cas9 technology [100]. Compared to random viral integration, the site-specific integration into TRAC locus of CAR transgene results in more uniform receptor expression, lower levels of tonic signaling and insertional-mutagenesis risk [57, 101].

Double-strand breaks may cause improper chromosomal rearrangements or large deletions. A more specific alternative, base editing, uses catalytically compromised Cas proteins which are coupled to either cytosine or adenine deaminases to alter just one base (C→T or A→G) without breaking both strands. This allows for more multiplex disruption of genes associated with inhibition or exhaustion with a much lower amount of double-strand-break related genomic instability as compared to classic nuclease editing [102]. However, base editing can still have an off-target effect. Low frequency of unwanted edits is seen due to the sensitivity of sequencing. For instance, there are additional “bystander” base changes in the editing window. Puzzling guide-less deamination’s have also occurred because of the deaminase. This has been seen in primary human T cells and HSCs. No adverse effects have been reported to date due to these “off-target” events that have occurred in the base-edited CAR-T trials reported to date. Newer and more precise base editors are being developed, and more sensitive detection assays are being developed to further decrease the editing window and off-target activity [103, 104]. The accuracy of prime editing is further enhanced through the use of a Cas9 nickase, an engineered reverse transcriptase and a prime editing guide RNA (pegRNA) to insert, delete and make all 12 possible base changes. These actions are without templates of donor DNA and without double-strand breaks. This is an important advantage for editing non-dividing, mature T lymphocytes [105]. Prior to the development of CRISPR/Cas9, other programmable nucleases, such as transcription activator-like effector nucleases (TALENs) and zinc-finger nucleases (ZFNs), were still clinically relevant and are used to disrupt endogenous TCR genes in some of the first universal CAR-T products to enter clinical trials [106].

The modern approach to editing provides a significant practical benefit, because many of the edits can be accomplished in one manufacturing run. For instance, a TRAC deletion could be combined with a B2M or HLA disruption, while simultaneously knocking out PD-1, LAG-3 or TIGIT, and making a site-specific CAR insertion [107]. This means it is possible to program resistance mechanisms; instead of having sequential changes in isolated genes, a random mutation of other genes. Future strategies could utilize transient RNA-based engineering for dynamically controlling CAR-T phenotype without permanent genomic engineering, or catalytically dead Cas in combination with transcriptional activators or repressors, and histone-modifying enzymes [108, 109]. These can be used in addition to normal editing to provide additional tunable and safer control of exhaustion, metabolism and cytokine production.

### Non-viral engineering and in vivo CAR-T cell generation

Despite the high production costs and the time required for clinically grade production, many clinically approved CAR-Ts are derived from the integration of γ-retroviral or lentiviral vectors, which provide for stable long-term expression. Further, these materials have a theoretical risk of insertional mutagenesis [110]. All these factors combined seriously restrict the global accessibility, particularly in low and middle-low income countries. A simpler approach is provided by non-viral engineering, which enables the introduction of plasmid DNA, messenger RNA (mRNA), CRISPR ribonucleoprotein complexes, and transposon systems into T cells with relative ease and cost-effectiveness in a multiparameter fashion [111]. Unlike standard vectors, Sleeping Beauty and PiggyBac are able to deliver larger genetic payloads such as complex synthetic circuits and cytokine cassettes to cells via stable integration (homologous, non-homologous, or both), and are also able to insert large pieces of mRNA into the genome without relying on viral particles, which may be advantageous for investigating new targets or regions of the genome where permanent modification introduces unpredictable safety concerns. The trends of using automated closed system bioreactors, which combine cell isolation, activation, transduction, and cell expansion into a single system, will further enhance reproducibility and reduce production timelines, now increasingly supported by AI-assisted in-process monitoring [112].

The biggest manufacturing innovation is the in vivo generation of CAR-T within the patient itself, which obviates both leukapheresis and ex vivo culture. Researchers are developing lentiviral or adeno associated viral vectors, as well as lipid nanoparticles (LNPs) carrying T-cell targeting ligands, for the convenient systemic delivery of genetic material encoding CAR to circulating lymphocytes [113]. It has been demonstrated that functional in vivo CAR-Ts have the potential to kill hematologic cancers in preclinical studies [114]. This strategy is under investigation in early Phase I trials for relapsed CD19 positive leukemia and multiple myeloma [115]. mRNA vaccines have proven to be very effective delivered with LNP. For the delivery of traditional therapeutic proteins, LNPs can be expected to significantly decrease the immunogenicity, simplify manufacturing and mitigate the need for repeat dosing. But selective and efficient targeting of circulating T cells is a challenging process which is still ongoing [19]. Long-term monitoring for off-target transduction as well as effective mitigation strategies are also important issues to solve first. If those are successful, non-viral and in vivo methods would potentially take the mill weeks-long nature of CAR-T manufacturing and make it more similar to an off-the-shelf biologic drug that can be administered when needed.

The generation of CAR by targeted viral vectors and targeted lipid nanoparticles (LNPs) engineering approaches results in a completely different in vivo CAR by working through a different mechanism and carrying different trade-offs. After integrating the CAR gene into the host genome, the CAR-T cells are fully functional after a single systemic injection with rapid and persistent clearance of target cells, without the need to make them ex-vivo with any significant cost. The mechanism of targeted LNPs is more advanced as compared to conventional LNPs that deliver only one type of drug. In doing so, they provide messenger RNA which is translated into CAR. This can consequently be used for a transient and self-limited CAR expression. Moreover, as a result of this delivery mechanism, there is no chance of genotoxicity resulting from viral integration. Thus, there is a requirement of repeat dosing for a sustained therapeutic effect. Similarly, newer LNP designs that instead package a DNA mini circle along with a transposase enzyme enable the generation of CAR-Ts that have a more stable gene transfer, but still a completely non-viral safety profile [116, 117]. Injectable hydrogels, porous scaffolds and nanoparticle “backpacks” attached to circulating T cells can also provide means for local and prolonged delivery of CAR-encoding vectors at the tumor site or an implanted depot. This may minimize systemic toxicity and production complexity in comparison to systemic delivery worldwide [118]. Researchers are making progress with different approaches to cellular therapy in which muscle, fat (particularly) or liver cells are modified in ways that are notable, such as mesenchymal stem cells, antigen specific T cells and other valuable cell types that could be put directly into patients. Few people are involved in working with minimally edited cells or gene modified cells [119].

In order for each platform to enable more straightforward manufacturing and improved efficiency, there are real challenges that must be overcome. Viral vectors are especially prone to off-target transduction of non-T cells, which can occur when manufacturing these extremely selected T cell therapeutics or after they are given. Not all malignant B cells will always be distinguishable from healthy targets before being given. This is especially significant because this challenge involves the uptake of the CAR displayed on the vector envelope by any target antigen expressing cells. Non-viral LNP systems would not be affected from this particular risk; however, they would have to surmount the naturally low endocytosis of nanoparticles by T cells. The latter needs to be equipped with targeting ligands like antibodies or nanobodies that bind to the surface receptor of the T cell to be able to deliver efficiently [117, 120]. It is becoming increasingly odd that more and more research groups today are dedicated to the generation of efficient CAR-T or CAR-NKs. Many European research groups demonstrate a lack of focus on the regulatory and safety-update aspects of allogeneic therapies. It remains unclear how many regulatory departments have prioritized activities aimed at clarifying these issues. If the in vivo generation of CARs can be achieved safely and delivered, then the need for leukapheresis and ex vivo culture for centralized manufacturing would be eliminated. This would provide one of the most straight-forward paths to a commercialized, low cost, off-the-shelf CAR-T product. This is already possible, per early clinical and preclinical data, in relapsed CD19-positive leukemia, multiple myeloma and even in non-cancer conditions like systemic lupus erythematosus [120, 121].

### Extending CAR-T therapy to solid tumors

A system-level engineering approach is needed to achieve the successful implementation of CAR-T therapy in solid tumors. This demands a number of barriers be optimized at once, instead of optimizing any one receptor property. Solid tumors are antigenically heterogeneous, have a dense stroma, abnormal vasculature, and strong immunosuppression.

Both the antigen heterogeneity inside a tumor and that between primary and metastatic tumor sites drives immune escape through means such as downregulation of the antigen and alternative splicing and selection of antigen-negative clones [122]. This has been addressed directly by the platforms discussed above such as dual-target, tandem, logic-gated, and synNotch platforms. Moreover, the analysis of genomics, transcriptomics and proteomics data using artificial intelligence is becoming widely used to discover combinations of antigens with the greatest tumor specificity [123]. This will help to save normal tissues as well. High interstitial pressure, dense extracellular matrix, mismatched chemokine signaling often result in poor infiltration. Engineering additional chemokine receptors (CCR2, CXCR1, CXCR2, CXCR3, CXCR6), expressing matrix degrading enzymes such as heparinase, and regional or intratumoral delivery are strategies being explored to improve infiltration [16, 39]. Spatial transcriptomics and multiplex imaging are uncovering that infiltration is not simply a numbers game, but rather a search for immune niches that are permissive [124].

Once inside the tumor, CAR-T cells face regulatory T cells, myeloid-derived suppressor cells, tumor-associated macrophages, TGF-β, IL-10, and PD-L1, which contribute to an immunosuppressive environment [125, 126]. In addition, hypoxia, nutrient deprivation, and adenosine signaling further hinders CAR-T proliferation and effector functions. Cytokine-armored, locally secreting IL-12, IL-15, IL-18 or IL-21 CAR-T cells can transform immunologically cold tumors into inflamed treatment responsive lesions [33]. Moreover, genome editing to remove PD-1 and TGF-β receptors, and synthetic circuits to deliver “local” checkpoint blockade maintain CAR-T cell function without systemic toxicity [127]. Hypoxia, nutrient competition, and lactate accumulation are the factors of hostile tumor metabolism that causes exhaustion. Therefore, next-generation CAR-T cells adopt metabolic engineering approaches, such as increased glucose and amino-acid uptake, mitochondrial biogenesis and removal of lipid receptors (e.g., CD36) to maintain CAR-T cell fitness in hostile conditions [8]. A biomarker guided patient selection approach is increasingly adopted; informed selection of the treatment, based on single cell sequencing, spatial multi-omics studies and AI predicting the resistance mechanisms, matches specific engineering strategies to individual biology, instead of a uniform treatment [123].

The initial clinical results are promising with these integrated approaches [128]. This approval was the first to be granted for a solid tumor, gastric cancer, for satri-cel, the first clinical evidence of the efficacy of next generation engineering strategies beyond hematological disease, and likely the first to help kickstart the global development of solid tumor CAR platforms.

### Combination strategies and multimodal CAR-T therapy

**Fig. 3:**
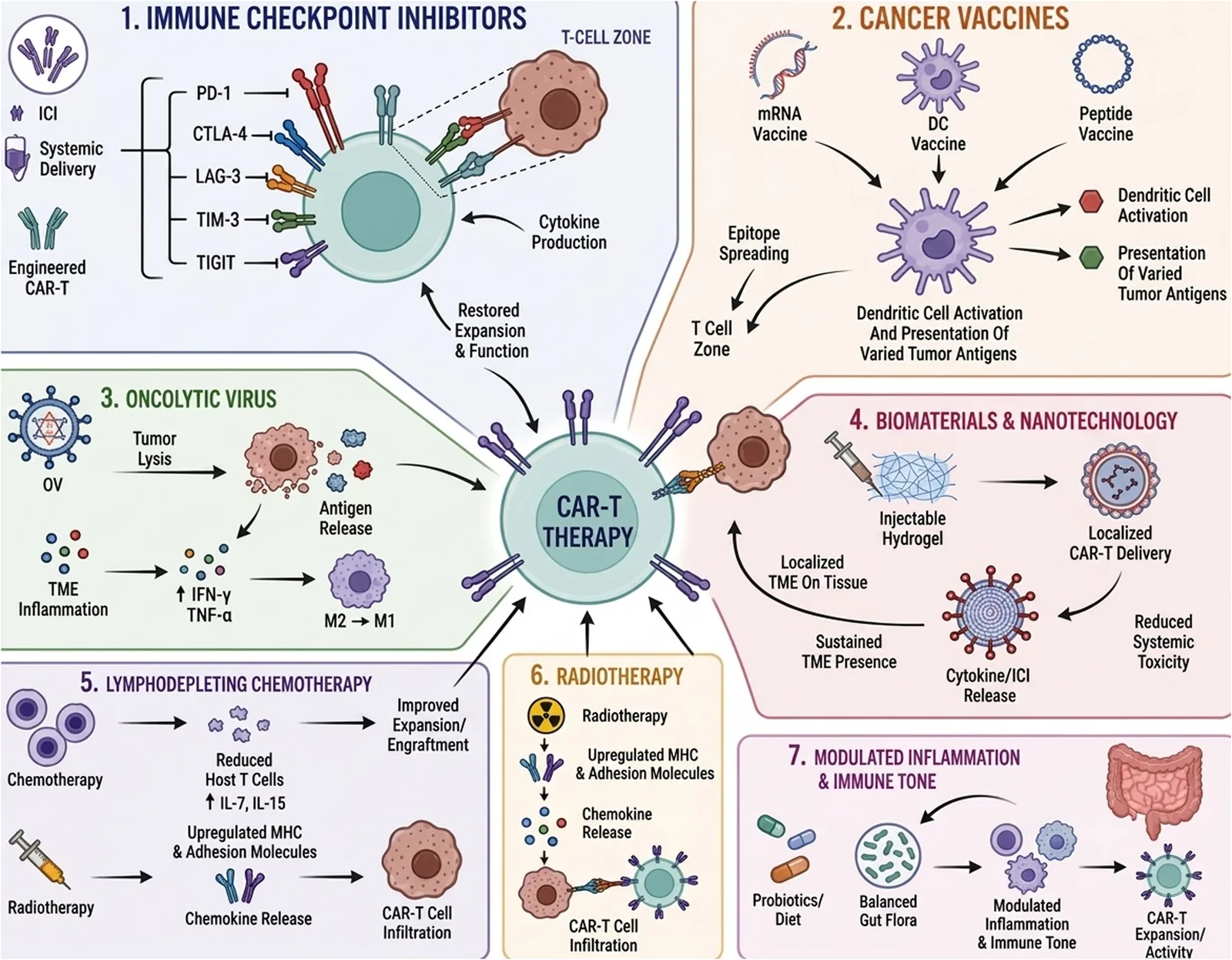
Multimodal approaches to enhance CAR-T cell therapy. Immune checkpoint inhibitors, cancer vaccines, oncolytic viruses, biomaterials and nanotechnology, lymphodepleting chemotherapy, radiotherapy, and gut microbiome modulation synergistically improve CAR-T cell persistence, tumor infiltration, antitumor activity, and therapeutic efficacy while overcoming immunosuppression and reducing toxicity

Advanced cancers (particularly solid tumors) have many different resistance mechanisms, which will require more than one change to overcome; hence, rational combination approaches are gaining ground. The focus of these approaches will be to harness biological synergy, rather than merely augment cytotoxic intensification. Figure 3 summarizes the major combination strategies that enhance CAR-T cell therapy by improving persistence, tumor infiltration, immune activation, and overall therapeutic efficacy.

Persistent stimulation of T cells by antigen increases inhibitory receptors such as PD-1, CTLA-4, LAG-3, TIM-3 and TIGIT. Systemic or local delivery of ICI to T cells or engineering of the CAR-T cells to lack the PD-1 receptor or express dominant-negative receptors locally can restore expansion, cytokine production and persistence [127]. In addition to these strategies, bispecific T-cell engagers (Bispecifics or BiTEs) and antibody-drug conjugates (ADCs) are now being used as part of CAR-T treatment sequences, and not just as alternatives to CAR-T. A bispecific antibody against BCMA or GPRC5D directed to the BCMA/CD3 and GPRC5D/CD3 bispecific T-cell idiotype has been reported to give at least comparable but perhaps superior response rates to standard bridging chemotherapy before CAR-T product manufacture in patients with multiple myeloma [129]. In B-cell lymphomas, bispecific antibodies and CAR-T cells are increasingly viewed as sequential or complementary options for the same patient, with growing clinical experience on how prior exposure to one modality affects the safety and efficacy of the other [130]. ADCs directed against tumor antigens are similarly being explored both as salvage therapy for antigen-low or antigen-negative relapses after CAR-T failure and as debulking agents before CAR-T infusion, potentially lowering the inflammatory tumor burden and reducing the risk of severe cytokine release syndrome during CAR-T expansion. Oncolytic viruses selectively kill tumor cells and induce immunity. They can convert cold tumors to inflammatory. Then these tumors can be targeted with CAR-T therapy, improving the benefits of cancer treatment [131, 132]. When radiotherapy is given before CAR-T infusion, the immunomodulatory effects of radiotherapy (such as MHC up-regulation, adhesion molecules, chemokines) are expected to be beneficial to the microenvironment for CAR-T cell infiltration [133]. In contrast, lympho-depleting chemotherapy standardly used in CAR T therapy is essential to reduce competing lymphocytes and boost homeostatic cytokines (IL-7, IL-15) that aid in CAR-T engraftment and expansion [134].

There are other strategies apart from immune modulation. Therapeutic cancer vaccines have a promise to enhance anticancer immune responses [135]. Using biomaterials and nanotechnology, it is also possible to inject CAR-T cells directly into the tumor tissue to achieve prolonged persistence, regulated and tumor-specific release of cytokines and checkpoint inhibitors and reduce systemic toxicity [136]. The microbiome of the gut has been found to be associated with CAR-T expansion, risk of toxicity, and outcome of treatment, and there is the potential for the microbiome to be modulated through diet, probiotics or fecal microbiota transplantation (FMT) that will become another avenue to improve response in the future [137]. Together, these multi-modal strategies represent a paradigm shift in the way CAR-T is viewed: a new, cellular product into a Precision Immunotherapy platform [138].

### Safety engineering and toxicity management

The most common serious toxicities of CAR-T therapy continues to remain CRS and ICANS, along with prolonged cytopenias (i.e., immune effector cell-associated hematotoxicity or ICAHT), infection, and macrophage activation syndrome (MAS), as well as on-target/off-tumor effects [139]. But due supportive care and standardized management guidelines, the mortality rate owing to treatment has substantially dropped. Toxicities, however, have hindered dose escalation, and the application of more potent CAR designs. Furthermore, next-generation studies are looking to prevent toxicities as part of the engineering process instead of treating them after they have been developed [127].

In addition to conventional treatment with tocilizumab and corticosteroid, potential approaches to decrease the overproduction of cytokines at their origin include GM-CSF knockout, tunable signaling domains, and drug-inducible activation [140]. Endothelial activation and blood-brain-barrier disruption are hallmarks of ICANS. The development of prediction models has been focused on imaging and biomarkers that are able to flag risk before the onset of neurological symptoms [141, 142]. Persistent hematotoxicity was previously thought to be due to myelosuppression. Now, this has been shown to be a sign of chronic inflammation and previous chemotherapy and marrow damage, in contrast. Clinical monitoring and supportive care are recommended to be guided by the use of composite risk scores, such as CAR-HEMATOTOX [143].

The next generation has a safety switch as a hallmark of its design. Infused CAR-T cells will undergo rapid and selective cell death in the inducible caspase-9 (iCasp9) system when the small molecule dimerizer is injected into the tumor, while others are truncated EGFRt, CD20 depletion and HSV-tk as elimination tags [144]. Any benefit that can be derived from the CAR will be lost if it is permanently destroyed and therefore it is advantageous to have options such as ON/OFF-switch CARs, degron-based regulation or adaptor-controlled platforms to transiently suppress activity without removal of the cells [145]. The more engineering safeguards added, a notion is to establish a safety profile that uses biomarker-based risk estimates. These estimates will be based on baseline cytokine levels, ferritin, tumor burden, single-cell or metabolomic signatures as well as AI based models that will help identify CAR-T patients at risk for severe CRS or ICANS prior to their development [142]. This transition will bring CAR-T safety from the reactive management to proactive individualized safety control [146].

### Regulatory and health-economic considerations

The pace at which the next-generation platforms will be available to patients will be directly affected primarily by regulatory processes and cost. The FDA’s Regenerative Medicine Advanced Therapy (RMAT) designation, Fast Track designation and Breakthrough Therapy designation, and the EMA’s PRIME scheme have all been granted various times to facilitate the development of promising cell and gene therapies [147, 148]. Several allogeneic and next-generation autologous CAR products reviewed here have been given at least one of these designations. Despite being promising, donor-based cell product regulatory approval took a long time as per early trial data indicated. As of this, there is not yet a fully-marketed allogeneic CAR-T product around the globe [149]. Furthermore, a clinically most advanced allogeneic off-the-shelf T-cell product (Epstein-Barr-virus-specific [non-CAR] donor T-cell therapy) already approved by the European Union since 2022 is not yet approved in the U.S. It was also the second time the FDA sent a second Complete Response Letter to the latter product in January 2026 over trial design and comparator issues, although it addressed such issues, it did not address efficacy or safety. The history serves as a case study for readers of this review of how accelerated progress in the preclinical and early phase of development of an allogeneic and next-generation CAR platform does not necessarily translate to a speedy, guaranteed path to approval [147, 148].

National regulations for cell and gene therapy are also rapidly changing and can significantly impact the rate at which new CAR platforms can get from the lab to the clinic in a specific country. On 1st May 2026, China’s main national regulation for clinical trials and clinical translation application of new biomedical technologies, such as cell and gene therapeutics, will impose more stringent institutional review and data reporting and safety monitoring requirements for early-phase human studies [150]. Future CAR platform developers will have to monitor this evolving regulatory environment along with the evolving science and investigators from various jurisdictions.

The cost is among the biggest challenges to universal access to CAR-T therapy, and it is a significant driver behind the increasing popularity of point of care (decentralized) manufacturing as a viable next-generation approach. Many academic point-of-care manufacturing schemes have reported a substantial cost reduction. For instance, in India, the treatment price of CD19 CAR-T cells manufactured using a closed automated manufacturing platform on-site is a lot less than what is offered by commercially-licensed products. Safety and response rates are reasonably similar to the published data [151]. In Spain, a commercial CD19 CAR-T product is reimbursed at a much higher cost compared to an academic CD19 CAR-T product [152]. These programs help to lower cost and shorten vein-to-vein time by eliminating the need to transfer cells between a centralized location and customer facilities [153]. However, as the scale-up of this model progresses, consistency of regulator grade quality control at several local academic manufacturing sites rather than single site is a significant operational challenge to overcome [154].

One important point to keep in mind when assessing the clinical success of CAR-T platforms is that results are not always comparable. Direct comparison of trials may be confounded even in published CAR-T products approved, due to variations in the criteria used with which the response was assessed, such as Lugano or RECIL criteria used in lymphoma, or International Myeloma Working Group (IMWG) criteria used in Multiple Myeloma. Large differences can also exist in the definitions of endpoints that are considered important: overall response rate, complete response, duration of response, MRD negativity, progression-free survival and overall survival. Moreover, the interpretation of reported outcomes can be impacted by differences in analysis populations, whether outcomes are assessed on an intention-to-treat or per-protocol basis, and the measurement of survival: from leukapheresis or CAR-T cell infusion. The degree of heterogenicity among studies is also significant, reflecting the different patient groups, disease burden, previous lines of therapy, bridging therapies, and follow-up durations. So, the results of single arm and early phase studies should not be viewed as evidence of superiority to existing CAR-T platforms. Comparisons will only be possible with certainty if RCTs or well-designed matched comparative studies with standardized clinical endpoints and adequate long-term follow-up are performed.

## Future perspectives

The CAR-T therapy can be defined to be changing from single antigen cytotoxic lymphocytes into programmable living medicines which integrate genome editing, synthetic biology, nanotechnology as well as computational design. The process starts with the selection of an antigen and receptor, and is carried out using AI. AI is also used to predict manufacturing outcomes and toxicity as well as in ‘digital twin’ simulations of patient-specific immune responses. The continued development of CAR-T cells’ sensing and response capabilities is possible with synthetic biology. This includes the ability to respond to signals including antigen density, oxygen tension and metabolic stress. Beyond oncology, CD19-directed CAR-T has delivered impressive outcomes in severe autoimmune disorders like systemic lupus erythematosus, multiple sclerosis, and systemic sclerosis, leading to the complete immune reset of patients, and other platforms such as CAR-NK, CAR-macrophages, CAR-Treg, and chimeric autoantibody receptor T (CAAR-T) cells are being investigated for fibrosis, chronic infection, and organ transplantation, respectively. The dependence on costly individual manufacturing is decreasing with the use of universal allogeneic products, iPSC-derived cells, non-viral engineering and in vivo generation. This has resulted in the shift of treatment towards true precision medicine with multi-omics, biomarker guided patient selection.

**Fig. 4:**
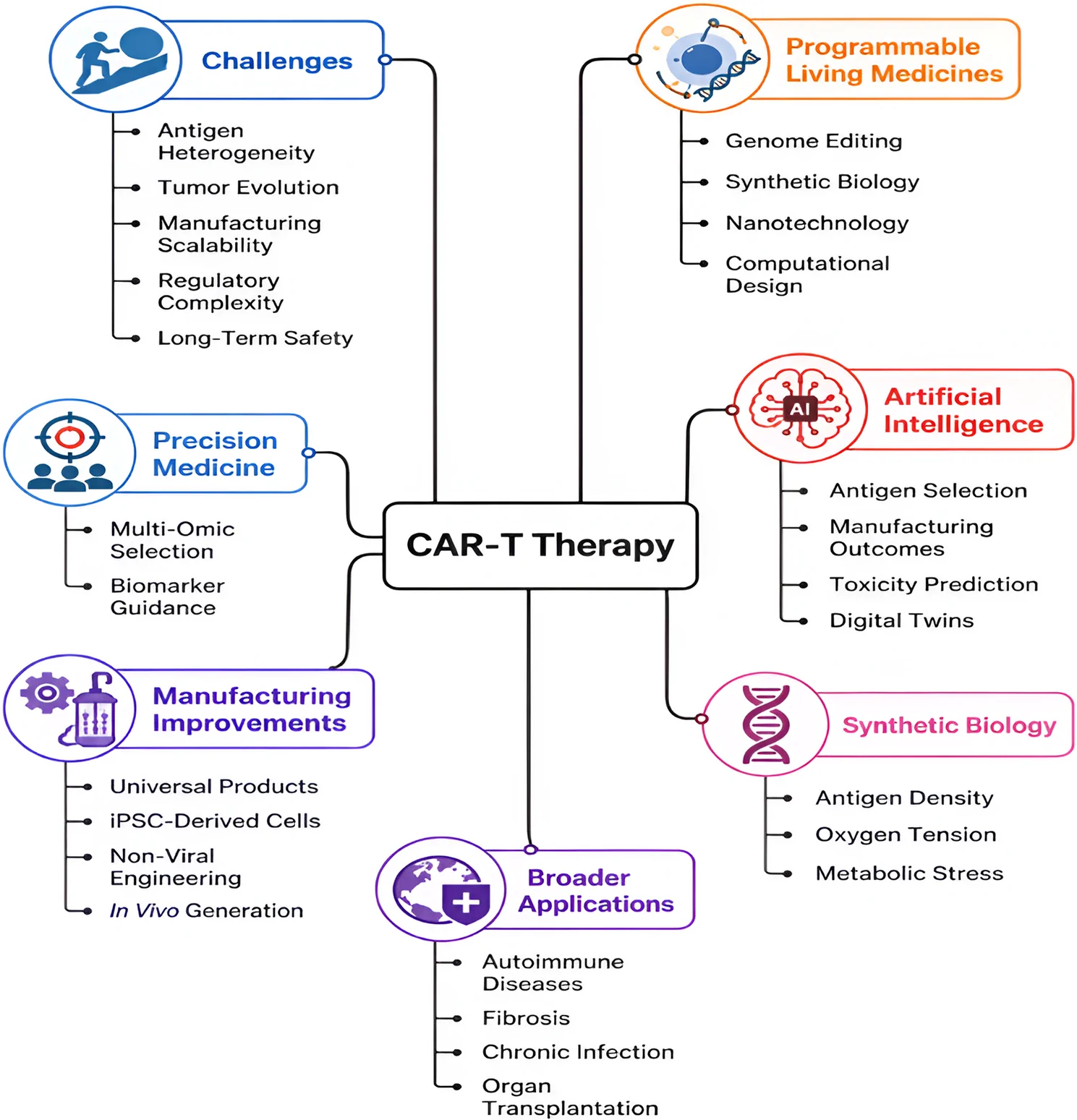
Future perspectives of CAR-T therapy

Substantial challenges remain. Antigen heterogeneity and tumor evolution and exhaustion remain limiting factors for the durability of responses, especially in solid tumors. Manufacturing scalability, regulatory complexity and affordability preclude global access. Ongoing long-term safety monitoring of multiplex genome editing and/or persistent immune modulation is necessary. With the evolution of programmable cellular therapeutics, the regulatory landscape needs to be updated accordingly. This includes the integration of synthetic biology, AI and self-governing biological decision making. The solution to these challenges does not depend on a single technology, but rather the continuous development of the technologies outlined in this review, namely genome editing, synthetic biology and precision medicine. Figure 4 summarizes future perspective.

## Conclusion

CAR-T cell therapy has revolutionized the treatment of blood cancers and is now systematically being re-engineered to overcome its key hurdles of antigen escape, T-cell exhaustion, treatment-related toxicity, limited solid-tumor efficacy and manufacturing challenges. New strategies like logic-gated, synNotch receptors, multiplex genome editing, universal platforms and in vivo platforms, combined with novel combination strategies like checkpoint inhibitors, oncolytic viruses, radiotherapy, and nanotechnology, are transforming CAR-T cells from simple antigen-targeting killers to programmable, safer, and more accessible therapies. As these approaches continue to advance, accumulating clinical experience, including the first approval of a solid tumor with satri-cel, next-generation CAR-T therapy is poised to become a key element in precision cellular medicine, with therapeutic activity not limited to cancer.

## Data Availability

No datasets were generated or analysed during the current study.

## Abbreviations

AI: Artificial intelligence
B2M: Beta-2 microglobulin
CAR: Chimeric antigen receptor
CAR-M: Chimeric antigen receptor macrophage
CAR-NK: Chimeric antigen receptor natural killer
CAR-T: Chimeric antigen receptor T cell
Cas9: CRISPR-associated protein 9
CRISPR: Clustered regularly interspaced short palindromic repeats
CRS: Cytokine release syndrome
CTLA-4: Cytotoxic T-lymphocyte-associated protein 4
DARPin: Designed ankyrin repeat protein
DSB: Double-strand break
GVHD: Graft-versus-host disease
HLA: Human leukocyte antigen
ICAHT: Immune effector cell-associated hematotoxicity
ICANS: Immune effector cell-associated neurotoxicity syndrome
IEC-HS: Immune effector cell-associated hemophagocytic syndrome
IFN-γ: Interferon-gamma
IL: Interleukin
iCasp9: Inducible caspase-9
LAG-3: Lymphocyte activation gene-3
MDSCs: Myeloid-derived suppressor cells
MHC: Major histocompatibility complex
mRNA: Messenger RNA
NK: Natural killer
PD-1: Programmed cell death protein-1
PD-L1: Programmed death-ligand 1
PDCD1: Programmed cell death protein 1 gene
pegRNA: Prime editing guide RNA
QC: Quality control
scFv: Single-chain variable fragment
synNotch: Synthetic Notch
TALENs: Transcription activator-like effector nucleases
TAMs: Tumor-associated macrophages
TGF-β: Transforming growth factor-beta
TIGIT: T-cell immunoreceptor with Ig and ITIM domains
TIM-3: T-cell immunoglobulin and mucin domain-containing protein-3
TME: Tumor microenvironment
TRAC: T-cell receptor alpha constant
TRUCKs: T cells redirected for universal cytokine-mediated killing
ZFNs: Zinc finger nucleases
